# The Association between Non-Alcoholic Fatty Liver Disease and Cardiovascular Risk in Children

**DOI:** 10.3390/children4070057

**Published:** 2017-07-07

**Authors:** Anna Di Sessa, Giuseppina Rosaria Umano, Emanuele Miraglia del Giudice

**Affiliations:** Department of Woman, Child and General and Specialized Surgery, University of Studies of Campania “Luigi Vanvitelli”, 80138 Napoli, Italy; giuseppinar.umano@libero.it (G.R.U.); emanuele.miraglia@unina2.it (E.M.D.G.)

**Keywords:** Non-Alcoholic Fatty Liver Disease, cardiovascular, risk, diseases, atherosclerosis, cardiac, abnormalities

## Abstract

The rising prevalence of childhood obesity in the past decades has made Non-Alcoholic Fatty Liver Disease (NAFLD) the most common cause of pediatric chronic liver disease worldwide. Currently, a growing body of evidence links NAFLD with cardiovascular disease (CVD) even at an early age. Data on the pediatric population have shown that NAFLD could represent an independent risk factor not only for cardiovascular events but also for early subclinical abnormalities in myocardial structure and function. Briefly, we review the current knowledge regarding the relationship between pediatric NAFLD and cardiovascular risk in an attempt to clarify our understanding of NAFLD as a possible cardiovascular risk factor in childhood.

## 1. Introduction

In the last 20 years, Non-Alcoholic Fatty Liver Disease (NAFLD) has become the major cause of chronic liver disease in childhood, as a result of an increased prevalence of pediatric obesity [1,2]. Although the natural history of pediatric NAFLD remains unclear, evidence suggests a possible link between NAFLD and a greater overall mortality than in the general population [3]. Furthermore, it has been widely recognized that NAFLD represents an independent risk factor for cardiovascular diseases (CVD) in adulthood, but this association is still debated in the pediatric population [4,5,6,7,8]. Most recent studies are conflicting and do not support an independent relation between NAFLD and CVD in pediatric patients. Despite that, some findings have shown that atherosclerosis is an early process beginning in childhood, as demonstrated by early subclinical atherosclerosis (flow-mediated vasodilatation (FMD), carotid artery intimal medial thickness (cIMT), arterial stiffness) and abnormalities in myocardial structure and function (left ventricular (LV) dysfunction, increased LV mass, and epicardial adipose tissue thickness (EAT)) observed in obese children and adolescents [4,7,8,9,10].

To date, the association between NAFLD and cardiovascular derangements has not been fully elucidated in children, but increased visceral adipose tissue and insulin resistance seem to play a critical role [8,10,11]. Increased levels of free fatty acids (FFAs) in children affected by NAFLD might explain—likely through myocardial lipid accumulation—the cardiac structural and functional alterations observed in these subjects [9,10]. Additionally, it could be hypothesized that the relationship might be related to the impaired systemic inflammatory pathway in the setting of NAFLD, contributing to cardiovascular changes—such as cardiac dysfunction and atherosclerosis—observed in this population [4,7,8]. In fact, the presence of a low-grade inflammatory state in those patients contributes to the release of several mediators, including C reactive protein (CRP), interleukin-6 (IL-6), tumor necrosis factor-alpha (TNF-alpha) and other cytokines that amplify this condition [8,10]. In particular, recent studies have focused on fibroblast growth factor 21 (FGF-21), a hepatoprotective protein with modulatory effects on glucose and insulin sensitivity, that shows a condition of resistance in subjects with NAFLD, with further worsening in the case of Non-Alcoholic Steatohepatitis (NASH) [11].

In order to stop this vicious circle, a comprehensive treatment of NAFLD (weight loss, lifestyle interventions, control of risk factors) is strongly recommended.

## 2. Atherosclerosis

Currently, NAFLD is considered as a hepatic manifestation of Metabolic Syndrome (MetS), which in turn has been well recognized as a highly atherogenic condition [11,12,13,14]. Studies have shown that atherosclerosis often begins in pediatric age, as demonstrated by cIMT and fatty streaks detected in the aorta and the coronary arteries in this population [12,15,16,17].

Although the relationship between obesity and atherosclerosis development has been studied in childhood, evidence regarding the association between NAFLD and atherosclerosis is scarce and conflicting at this age [18,19,20]. Indeed, in contrast to the adult population, pediatric studies have shown no evidence of heterogeneity and the link between NAFLD and cardiac abnormalities in children is yet to be proven. To date, in fact, there is a lack of adequate (e.g., long-term longitudinal studies) or properly adjusted (e.g., gender, race, pubertal age, insulin resistance or other important cardiovascular risk factors) studies in pediatric patients. Some studies have shown an independent association between NAFLD and FMD and increased cIMT, considered as markers of subclinical atherosclerosis [3,8,9,10,12]. To the best of our knowledge, potential pathophysiological mechanisms by which NAFLD may cause atherogenesis are unclear, but increased visceral adipose tissue (particularly periaortic fat thickness (PAFT) a subtype of perivascular fat with local pathogenic action on blood vessels), insulin resistance, disturbance in lipoprotein metabolism (apolipoprotein B), and subsequently, release of inflammatory cytokines from the liver can play an important role.

Moreover, recent published data have shown that children with NAFLD are at increased risk of CVD, as demonstrated by various cardiac outcomes such as left ventricular mass index, cIMT, coronary parameters and endothelium-dependent flow-mediated dilatation [7,8,9,10,16,17,18,19,20,21].

Eklioğlu et al. reported that obese children with NAFLD had increased PAFT compared to those without NAFLD [22]. Moreover, in the NAFLD group, waist circumference was found to be the only parameter among all other clinical and laboratory predicting PAFT. Consequently, these findings suggested that the NAFLD group may present a higher adverse cardiovascular risk profile—due to the atherogenic role of PAFT—than those without NAFLD. Thus, it is probable that NAFLD and atherosclerosis share a similar pathogenesis, with a potential reciprocal influence between the liver and blood vessels (“liver–vessel axis”).

Gökçe et al. showed a positive correlation between cIMT and insulin resistance, MetS and triglycerides (TG) levels in children with NAFLD [23].

In the Bogalusa heart study in children, it has been demonstrated that high levels of total cholesterol, low-density lipoprotein cholesterol (LDL-c), triglycerides, and low concentrations of high-density lipoprotein cholesterol (HDL-c) are significantly associated with the extent of intimal surface involved in atherosclerotic lesions [24]. In particular, the TG/HDL-c ratio has been well recognized as a strong predictor of MetS and CVD. Most recent pediatric studies suggest the possible role of increased cIMT as a marker of structural vascular changes and metabolic derangements in obese children with NAFLD [23,24,25,26,27].

Moreover, the degree of liver steatosis may represent an independent predictor of the proatherogenic lipid profile—as observed by Nobili et al.—and its positive association with increased cIMT suggests that it may be considered as a possible prominent risk factor of future cardiovascular disease [25].

On the other hand, some pediatric studies reported no association between NAFLD and CVD [6,8]. In a cross-sectional study conducted on 78 obese children, who underwent a clinical and carotid ultrasound assessment (cIMT, arterial stiffness), no early atherosclerosis changes were found in patients with NAFLD [6].

However, further studies are needed to investigate the relationship between NAFLD and atherosclerosis in children.

## 3. Cardiac Dysfunction

In the pediatric population, data about the relationship between NAFLD and both cardiac function and geometry are scarce [26,27,28,29,30]. Sert et al. reported an impaired diastolic function and an increased LV mass in adolescents with NAFLD compared to both healthy controls and obese adolescents without NAFLD [26]. LV mass was positively correlated with a Homeostasis model assessment of insulin resistance (HOMA) and alanine aminotransferase (ALT) levels. However, the design of the study (cross-sectional study) did not allow us to determine whether liver steatosis caused cardiac damage (increased cIMT, LV remodeling) [26]. Recent findings from Alp et al. confirmed significant echocardiographic alterations in the pediatric NAFLD group, whose EAT was directly related to the severity of fatty liver [27]. Moreover, intriguing data from a group of 50 children with biopsy-proven hepatosteatosis suggest that children with NAFLD have cardiac damage, and that the liver itself could affect the cardiac geometry [28].

This is the hot spot of the scientific debate. More studies are needed to clarify the role of liver steatosis in worsening cardiac structure and function. Interestingly, Pacifico et al.—confirming the presence of cardiac abnormalities (LV structural and functional changes) in obese children and mostly in the NAFLD group—have found that cardiac alterations were more pronounced in children with NASH [29]. This finding suggests a possible role of hepatosteatosis in increasing the release of FFAs by inducing epicardial fat deposition, a depot metabolically active that could have a paracrine interaction with myocardium [29].

However, a further possible pathophysiological mechanism regarding the role of NAFLD in cardiovascular outcomes could be represented by the worsening of the insulin sensitivity shown in obese children with NAFLD, as demonstrated by Singh et al. [30]. In fact, these patients showed higher C-peptide plasma levels and a lower Disposition Index (DI) during an oral glucose tolerance test (OGTT). The lowering of DI was negatively related to the intrahepatic fat content. In addition, in this group, higher systolic and diastolic blood pressure levels and an abnormal cardiac functionality (decreased LV global longitudinal systolic strain and early diastolic rates) were found compared to non-NAFLD patients, suggesting a possible role of NAFLD as an early marker of cardiac dysfunction [30].

## 4. Conclusions

NAFLD in childhood is an evolving condition, potentially affecting many extra-hepatic organs; it requires a multidisciplinary approach to its treatment.

NAFLD and cardiovascular changes are closely linked in adulthood, but at the present time this relationship in childhood is still discussed and its pathophysiology is not yet fully understood. Although it has not been widely demonstrated, recent data have shown that NAFLD could be considered as a marker of subclinical atherosclerosis as well as a strong cardiovascular risk factor even at a very early age.

Given the findings from the available data it has been determined that both subclinical atherosclerosis and cardiac dysfunction in children are a dynamic process due to the plasticity of the cardiovascular system at this age. In fact, prevention and treatment of NAFLD—if applied early—may play a crucial role in avoiding not only end-stage liver disease but also CVD.

Furthermore, long-term longitudinal studies are needed to clarify the effective role of pediatric NAFLD in influencing cardiovascular outcomes.
